# A Note on the Improper Management of Epileptics

**Published:** 1906-06

**Authors:** Marc Ray Hughes

**Affiliations:** St. Louis, Mo.; Professor of Mental and Nervous Diseases. Barnes University (Med. Dept.); Professor of Criminal Anthropology, Benton College of Law, etc.


### TEXAS MEDICAL JOURNAL.

ESTABLISHED JULY, 1885.

PUBLISHED MONTHLY.—SUBSCRIPTION $1.00 A YEAR.

VOL. XXI. AUSTIN, JUNE, 1906. No. 12.

The publisher is not responsible for the views of contributors.

For Texas Medical Journal.

### A Note on The Improper Management of Epileptics.

BY MARC RAY HUGHES, M. D., ST. LOUIS, MO.

Professor of Mental and Nervous Diseases. Barnes University (Med. Dept.); Professor of Criminal Anthropology, Benton College of Law, etc.

Not many years have elapsed since epilepsia was listed in the category of incurable diseases, in fact, most people now believe it to be a disease never entirely eradicated by treatment. There are many reasons why it should be now classed as a curable malady. Like all diseases, the more we study the etiology and pathology, the better we become acquainted and are more capable of producing results from our treatments.

Heretofore scarcely anything was known regarding the pathology of this disease. Now we know that there is a distention of the ventricles of the brain and a decided contraction of the arterioles of the cerebral cortex, though each stage of the disease has a separate and, in a manner, a distinct pathology of its own. In the grand mal type there is more fluid in the ventricles and more vasomotor disturbance due to pressure. There is more of the alternating state of irritation and paralysis of the vasomotor system of the brain and allied nervous system, especially at the beginning of the attack.

That is the first step in the pathology of grand mal. There are two other steps and stages in this variety of the disease that it is not necessary to mention here, but suffice it to say that each stage and each pathology of that stage can be produced artificially in animals and studied. A trauma, a blood toxine and autotoxines are producers of the disease; likewise the intravenous injection of absinthe, ammonium carbonate, alcohol, camphor, etc.; also irritation to the vasomotor brain blood supply and superinduction of ventricular fluid by galvanism and Faradism.

This disease is one that should be handled in the carefullest manner. One reason why it is so widely classed as an incurable disease is the fact that the treatment has been overdone at the wrong time or not carried on far enough at the proper time. The use of electricity has been greatly abused; likewise the bromides. Electricity is capable of producing epilepsy according to the area of brain covered by the flow of the current and the way the poles or electrodes are placed. The bromides are not drugs to be given together, and results are much better when given separately.

It is not possible to effect a cure by the use of bromides alone—not by any means—but many think so. The patient, in the first place, in many instances is not carefully examined, is placed upon bromide and electric treatment at once, as soon as the diagnosis is made, without respect or regard to the exact mental phenomena that are taking place within the structures of the brain or that particular time, or without knowing or understanding why that condition is present. Naturally enough, the results are negative, and that is the first reason why it is considered incurable. The second reason is because pains are not taken in the first and subsequent examinations to ascertain what mental phenomena are taking place within the cortex and giving rise to that particular stage of the disease at that time and what is likely to follow. The third reason is that most practitioners believe the treatment of all epileptics to be directed alike, therefore, the examinations are shallow, and the patient does not recover.

